# Vertebral artery hypoplasia as an independent risk factor of posterior circulation atherosclerosis and ischemic stroke

**DOI:** 10.1097/MD.0000000000027280

**Published:** 2021-09-24

**Authors:** Yasemin Dinç, Rifat Özpar, Büşra Emir, Bahattin Hakyemez, Mustafa Bakar

**Affiliations:** a Uludağ University Medicine Faculty, Department of Neurology, Turkey; b Uludağ University Medicine Faculty, Department of Radiology, Turkey; c Izmir Katip Celebi University, Department of Biostatistic, Turkey.

**Keywords:** acute ischemic stroke, cerebroservical athereosclerotic stenosis, vertebral artery hypoplasia

## Abstract

Vertebral artery hypoplasia (VAH) is a frequent anatomical variation of vertebral arteries, with emerging evidence suggesting that it contributes to posterior circulation ischemia. However, the relationship between VAH and ischemic stroke remains unknown. Hence, this study aimed to determine the prevalence of VAH in patients diagnosed with acute ischemic stroke who were followed up in a neurology clinic and to determine if it can potentially be a risk factor for atherosclerotic stenosis in vertebrobasilar circulation.

This retrospective study included 609 patients diagnosed with acute ischemic stroke between January 1, 2019 and January 1, 2020. Demographic of patients, risk factors, radiological and clinical characteristics were evaluated.

Posterior circulation was very common in patients with VAH, and the most common locations of atherosclerotic stenosis were V1 and V4 segments of the vertebral artery and the middle segment of basilar artery. Analysis of the risk factors for atherosclerotic stenosis in patients with posterior circulation acute ischemic stroke suggested that VAH was an independent risk factor.

Findings of the study suggest that VAH pre-disposes atherosclerotic stenosis in vertebrobasilar circulation, although its mechanism remains unknown. Hemodynamic parameters associated with atherosclerosis could not be measured in vivo. Thus, to better understand the underlying mechanism, conducting studies that examine blood flow parameters with high-resolution magnetic resonance angiography in patients diagnosed with acute cerebral ischemia patients with VAH is warranted.

## 1 Introduction

Vertebral arteries are the primary source of arterial supply to structures of the posterior fossa.^[1]^ Vertebral artery hypoplasia (VAH) is a frequent anatomical variation of vertebral arteries, and due to its high prevalence, the clinical influence of VAH has been appraised.^[2,3]^ Evidence suggested that VAH can cause posterior circulation ischemia (PCI), although the underlying mechanism remains uncertain.^[2–4]^

VAH is defined as vertebral artery diameter with a difference of more than 2 mm (V4 segment).^[5]^ VAH asymmetry has been identified by several study protocols; however, there are limited studies concerning literature that persistently describes asymmetry or hypoplasia and determines the clinical importance of VAH.^[6,7]^ There are limited study protocols considering the mechanism of cerebral ischemia in patients with VAH. Nonetheless, whether VAH is an independent risk factor for PCI or a risk factor for PCI only when combined with other atherosclerotic risk factors and the mechanism of PCI remains uncertain. Is the relationship between VAH and PCI related to the patient's sex, age, or additional risk factors? Ischemic cerebrovascular disease is a heterogeneous group of diseases caused by different mechanisms.

This study aimed to determine the prevalence of VAH in patients diagnosed with acute ischemic stroke who were followed up in a neurology clinic and to determine if VAH is a risk factor for vertebrobasilar circulation atherosclerotic stenosis.

## 2 Materials and methods

This retrospective study was conducted to determine the relationship between VAH and vertebrobasilar circulation atherosclerotic stenosis in patients diagnosed with acute ischemic stroke in a neurology clinic at the Uludag University. The study was approved by the Ethics Committee of Uludag University, Faculty of Medicine on September 18, 2019 (2019-15/10). Patient approval was not required for this study.

### 2.1 Patients

The study included 609 patients diagnosed with acute ischemic stroke following neurological examination and neuroimaging between January 1, 2019 and January 1, 2020 at the Uludag University Faculty of Medicine, Department of Emergency. Of the 609, 241 were females (39.6%) and 368 males (60.4%). The patients’ age ranged from 28 to 93 years, with a mean age of 66 (15 ± 11.77) years.

The inclusion criteria were as follows: admission to the emergency department of Uludag University within 24 hours following focal neurologic deficit, acute ischemic stroke diagnosis following neuroimaging, determined etiologic factor for the stroke and a regular 3-month follow-up following a stroke at the neurology clinic of Uludag University. The exclusion criteria were as follows: increased urea-creatinine level, renal pathologies, patients followed up in another study, patients where cerebral-neck computerized tomography angiography (CTA) was not possible, incomplete examination to enlighten the etiology of stroke.

All patients were examined by a neurologist and then hospitalized after considering for initial cranial computed tomography (CT) and appropriate treatment was initiated. To determine the etiology of stroke, cerebral-neck CTA, cranial magnetic resonance imaging, control cranial CT, electrocardiogram, echocardiogram, 24-hour rhythm holter and, if necessary, diagnostic cerebral angiogram were performed.

### 2.2 Data collection

Data including age, sex, symptom initiation time, medical history (history of stroke), hypertension (HT), coronary artery disease, diabetes mellitus (DM), cardiac failure, atrial fibrillation (AF), valvular disease, liver or kidney failure, and medication use were recorded in the hospital's registry and patients’ epicrisis. Of 659 patients diagnosed with acute ischemic stroke who were followed up in our neurology clinic, 609 were included in the study in line with the inclusion and exclusion criteria. Of the 50 patients excluded from the study, cerebral-neck CTA could not be performed in 27 patients, stroke etiology could not be determined in 10 patients due to incomplete evaluation, and the remaining 13 patients had irregular follow-ups at the clinic of neurology of Uludag University for 3 months following stroke. The neurologist used the Trial of Org 10172 in Acute Stroke Treatment (TOAST) classification to determine the etiologies of stroke. The radiologist evaluated the CT angiographies and the diameter difference of both vertebral arteries was measured in the V4 segment, and those more than 2 mm were diagnosed with VAH.^[5]^

### 2.3 CTA protocol

CTA examinations were conducted using 128-section Somatom Definition AS+ (Siemens, Erlangen, Germany) multidetector CT device. Using a topogram, images in the coronal plane were obtained. Non-contrast images were also obtained in the axial plane from the aortic arch to the vertex. Next, a low-dose axial slice (pre-monitoring) was taken from the aortic arch level. A region-of-interest was placed in the arch lumen for the bolus tracking method on this slice. A non-ionic iodinated contrast medium (iohexol 300 mg) was injected into the antecubital venous system, using an automatic injection device with a dose of 1.5 mg/kg at the rate of 5 mL/s and saline flush with the same dose. During the injection, low-dose axial slices repeated every second were obtained from the same level with pre-monitoring. Bolus tracking was performed continuously with Hounsfield Unit measurements on monitoring slices. When the Hounsfield Unit value exceeded 70 in region-of-interest, arterial phase images were obtained with the same slice thickness, number, and plane characteristics of non-contrast CT. Furthermore, digital subtraction of non-contrast images from arterial phase images was performed using NeuroDSA module in the Syngo CT Workplace (Siemens, Erlangen, Germany) program for bone removal and to obtain CTA Source Images. In non-contrast and arterial phase images, section thickness was 0.6 mm with no gap, dose 120 kV and reconstruction kernel H10f. Tube current was determined by attenuation-based current tube modulation (CARE Dose). Volumetric CT dose index (CTDIVol) values were 0.09 mGy in topogram, 20.88 in non-contrast images, 2.53 in pre-monitoring, 5.06 in monitoring, and 26.97 in arterial phase images.

### 2.4 Image evaluation

CTA Source Images was used for image evaluation. The anterior circulation describes the cerebral areas supplied by the branches of the internal carotid arteries.^[8]^ The posterior circulation consists of 2 vertebral arteries, basilar artery, 2 posterior cerebral arteries, and their branches.^[9]^ The vertebral artery is divided into 4 segments: V1, V2, V3, and V4. V1 extends from origin to the C6 vertebral foramen entrance, V2 extends from the C6 vertebral foramen to the C2 foramen exit, V3 extends from the C2 vertebral foramen exit to the dura mater, and V4 extends from the dura mater to the vertebrobasilar junction.^[10]^ The basilar artery was evaluated in 3 segments: the proximal segment, which extends from the vertebrobasilar junction to the origin of anterior inferior cerebellar arteries; the middle segment, which extends from the origin of the anterior inferior cerebellar arteries to the origin of the superior cerebellar arteries; and the distal segment, which extends from the origin of superior cerebellar arteries to its distal end.^[11]^

The presence of atherosclerotic stenosis in the intra-cranial vessels of the patients was evaluated by the radiologist according to the NASCET method. The vertebral and basilar arteries were recorded in the segments indicated with bt angio whether there was atherosclerotic stenosis. Patients were called for control 1 month after discharge, and modified Rankin scale was evaluated at the neurology outpatient clinic 3 months later to determine the clinical outcome

### 2.5 Statistical analysis

Radiological, demographic, and clinical data of patients were compared. Statistical analysis was conducted using the IBM SPSS Statistics 25.0 package program (IBM Corp., Armonk, New York). Kolmogorov-Smirnov test, a histogram, and Q-Q plot were implemented to determine data normality. After describing the normality, means and standard deviations, or medians (25%–75% quartiles), they were subjected to continuous variables. Frequencies and percentages were considered as categorical variables. Levene test was applied to determine the variance homogeneity. A 2-sided independent sample *t* test or a 2-sided Mann–Whitney *U* test was used to analyze the differences between groups for continuous variables. A 2-sided Fisher exact, Pearson chi-square, and continuity correction test for (2 × 2) or (r × c) tables were used to analyze the differences between the group for categorical variables. Binary logistic regression analysis was applied to determine the risk factors of the posterior circulation atherosclerotic stenosis. A *P* value <.05 was considered statistically significant.

## 3 Results

VAH was prevalent in 37% of the patients diagnosed with acute ischemic stroke. Of 225 patients with VAH, 133 were right-sided, while the remaining 92 were left-sided. Right-sided VAH was observed in 20.3% of the patients as compared to the left at 9.8%. In addition, VAH was more frequently observed in men (21.8%) than in women (15.1%) (*P* = .01).

Table 1 shows the comparison between patients with VAH and without VAH, including the clinical, demographic, and radiologic characteristics. Table 1 suggests that patients with VAH were more likely to develop PCI (*P* < .001) and atherosclerotic stenosis in the posterior circulation (*P* < .001). Factors such as being male (*P* = .01) and smoking (*P* = .008) were frequently associated with VAH. Other clinical characteristics such as age, HT, DM, coroner artery disease, modified Rankin scale, good clinical outcome, AF, and low-density lipoprotein (LDL) level showed no statistically significant difference (*P* > .05).

**Table 1:** The comparison of patients having vertebral artery hypoplasia and not and clinical, demographic, and radiologic properties.

	Patients with VAH (n = 225)	Patients without VAH (n = 384)	*P* value
Age^∗^ mean ± SD	66.14 ± 10.85	66.15 ± 12.29	.737
Sex (male gender)^†^	151 (%67.1)	74 (%19.2)	**.01**
Hypertension^†^	177 (%78.6)	295 (%76.8)	.599
Diabetes mellitus^†^	90 (%40)	149 (%38.8)	.770
Smoking^†^	103 (%45.8)	134 (%34.9)	**.008**
Coroner artery disease^†^	45 (%20)	81 (%21)	.748
LDL level^∗^ mean ± SD	128 ± 42.77	122.31 ± 35.94	.109
Stroke recurrence^†^	49 (%21.7)	52 (%23.1)	**.012**
Atrial fibrilation^†^	58 (%25.7)	127 (%33)	.059
Anterior circulation stroke^†^	142 (%63.1)	293 (%76.3)	**.001**
Posterior circulation stroke^†^	98 (%43.5)	98 (%25.5)	**<.001**
Anterior circulation atherosclerotic stenosis^†^	105 (%46.6)	117 (%30.4)	**.001**
Posterior circulation atherosclerotic stenosis^†^	82 (%36.4)	19 (%4)	**<.001**
Craniocervical atherosclerotic stenosis^†^	145 (%64.4)	134 (%34.8)	**<.001**
Clinical outcome (good clinical outcome)^†^	161 (%71.5)	283 (%73.6)	.566
TOAST stroke etiology^†^			
Large artery atherosclerosis	111 (%49.3)	96 (%25)	**<.001**
Cardioaortic	57 (%25.3)	137 (%35.6)	
Small-vessel occlusion,	30 (%13.3)	81 (%36)	
Other determined etiology	3 (%1)	17 (%4.4)	
Undetermined etiology	24 (%10.6)	53 (%13.8)	
The stenotic segment of vertebral artery			
V1^†^	28 (%12.4)	8 (%0.2)	**<.001**
V2^†^	13 (%5.7)	4 (%0.1)	**.001**
V3^†^	9 (%4)	2 (%0.05)	**.002**
V4^†^	45 (%20)	11 (%2.8)	**<.001**
The stenotic segment of basilary artery			
Proximal segment^†^	4 (%1.7)	0 (%0)	**.005**
Middle segment^†^	8 (%3.4)	1 (%0.4)	**.004**
Rostral segment^†^	3 (%1.3)	1 (%0.4)	.120

LDL = low-density lipoprotein, TOAST = Trial of Org 10172 in Acute Stroke Treatment, VAH = vertebral artery hypoplasia.

∗ Mann–Whitney *U* test.

† Pearson chi-square/continuity correction/Fisher exact test.

The patients were categorized according to TOAST classification; the etiology of stroke in 207 patients was large artery atherosclerosis, cardioembolic in 194 patients, small vessel occlusion in 111 patients, other determined etiologies in 20 patients, and undetermined etiologies in 77 patients. A significant difference was observed between the etiology of stroke and VAH (*P* < .001). Furthermore, 101 (17%) patients were diagnosed with atherosclerotic stenosis in the posterior circulation. Clinical, demographic, and radiological characteristics were compared, and a statistically significant relationship was observed between atherosclerotic stenosis in the posterior circulation and sex (0.026), DM (0.037), AF (*P* < .001), VAH (*P* < .001), anterior circulation stroke (*P* < .001), PCI (*P* < .001), and TOAST stroke etiology (*P* < .001). However, as indicated in Table 2, there was no statistically significant relationship between atherosclerotic stenosis in the posterior circulation and age, HT, smoking, coronary artery disease, LDL level, stroke recurrence, clinical outcomes, and atherosclerotic stenosis in the anterior circulation (*P* > .05).

**Table 2:** The comparison of patients having posterior circulation atherosclerotic stenosis and not and clinical, demographic, and radiologic properties.

	Patients with posterior circulation atherosclerotic stenosis (n = 101)	Patients without posterior circulation atherosclerotic stenosis (n = 508)	*P* value
Age^∗^ mean ± SD	66.89 ± 10.68	661 ± 11.98	.555
Sex (male gender)^†^	71 (%70.2)	297 (%58.4)	**.026**
Hypertension^†^	85 (%84.2)	16 (%3.1)	.105
Diabetes mellitus^†^	49 (%48.5)	190 (%37.4)	**.037**
Smoking^†^	47 (%46.5)	190 (%37.4)	.086
Coroner artery disease^†^	23 (%22.7)	103 (%20.2)	
LDL level^∗^ mean ± SD	130.481 ± 48.78	123.201 ± 36.26	.204
Stroke recurrence^†^	22 (%21.7)	79 (%15.5)	.085
Atrial fibrilation^†^	17 (%16.8)	168 (%33)	**.001**
VAH^†^	82 (%81.1)	143 (%28.1)	**<.001**
Anterior circulation stroke^†^	43 (%42.5)	393 (%77.3)	**<.001**
Posterior circulation stroke^†^	66 (%65.3)	130 (%25.5)	**<.001**
Anterior circulation atherosclerotic stenosis^†^	50 (%49.4)	260 (%51)	.058
Craniocervical atherosclerotic stenosis^†^	101 (100)	181 (%35.6)	**<.001**
Clinical outcome (good clinical outcome)^†^	74 (%73.3)	370 (%72.8)	.929
TOAST stroke etiology^†^			
Large artery atherosclerosis	65 (%64.3)	142 (%27.9)	**<.001**
Cardioaortic	19 (%18.8)	175 (%34.4)	
Small-vessel occlusion,	8 (%7.9)	103 (%20.2)	
Other determined etiology	2 (%1.9)	18 (%3.5)	
Undetermined etiology	7 (%6.9)	70 (%13.7)	

VAH = vertebral artery hypoplasia, LDL = low-density lipoprotein, TOAST = Trial of Org 10172 in Acute Stroke Treatment.

∗ Mann–Whitney *U* test.

† Pearson chi-square/continuity correction/Fisher exact test.

As shown in Table 3, significant variables for VAH were analyzed using binary logistic regression, and the most significant variable noted was atherosclerotic stenosis in the posterior circulation (*P* < .001). Furthermore, significant variables for atherosclerotic stenosis in the posterior circulation were analyzed using binary logistic regression, and VAH was noted as the most significant variable (*P* < .001) (Table 4). When analyzed by segmenting the posterior circulation, the most common segments of atherosclerotic stenosis in patients with VAH were V1 (*P* < .001), V4 (*P* < .001), and the middle segment of the basilar artery (*P* = .005) (Table 5).

**Table 3:** Evaluation of significant variables of vertebral artery hypoplasia using binary logistic regression.

			%95 CI for EXP(B)	
	*P* value	Odds ratio	Lower	Upper
Sex Ref: male gender vs female gender	.252	1.265	0.846	1.892
Smoking Ref: present vs absent	.679	1.093	0.717	1.668
Stroke recurrence Ref: present vs absent	.067	1.573	0.969	2.554
Anterior circulation atherosclerosis Ref: present vs absent	.748	1.085	0.661	1.781
Posterior circulation atherosclerosis Ref: present vs absent	**<.001**	9.054	5.210	15.736
TOAST stroke classification				
Large artery atherosclerosis Ref: present vs absent	**.033**	1.756	1.047	2.944

Significance of the model <.001. Significant variables are shown in bold. Ref = references, TOAST = Trial of Org 10172 in Acute Stroke Treatment, vs = versus.

**Table 4:** Evaluation of significant variables of posterior circulation atherosclerotic stenosis using binary logistic regression.

			%95 CI for EXP(B)	
	*P* value	Odds ratio	Lower	Upper
Sex Ref: male gender vs female gender	.238	1.369	0.813	2.307
Diabetes mellitus Ref: present vs absent	.054	1.612	0.991	2.620
Vertebral artery hypoplasia Ref: present vs absent	**<.001**	10.804	6.273	18.606
Atrial fibrilation Ref: present vs absent	**.018**	0.479	0.260	0.882
Anterior circulation atherosclerotic stenosis Ref: present vs absent	.702	1.101	0.673	1.802

Significance of the model <.001. Significant variables are shown in bold. Ref = references, vs = versus.

**Table 5:** Evaluation of vertebral artery hypoplasia and stenosis segments of the basilar and vertebral artery using binary logistic regression.

			%95 CI for EXP(B)	
	*P* value	Odds ratio	Lower	Upper
Vertebral artery V1 segments Ref: present vs absent	<.001	7.004	2.478	19.793
Vertebral artery V2 segments Ref: present vs absent	.836	0.849	0.181	3.989
Vertebral artery V3 segments Ref: present vs absent	.910	1.110	0.180	6.840
Vertebral artery V4 segments Ref: present vs absent	**<.001**	8.13	3.959	16.721
Basillary artery proximal segments Ref: present vs absent	.999	18.7	0.000	–
Basillary artery middle segments Ref: present vs absent	**.005**	19.7	2.444	158.978
Basillary artery rostral segments Ref: present vs absent	.308	3.5	0.390	41.092

Significance of the model <.001. Significant variables are shown in bold. Ref = references, vs = versus.

## 4 Discussion

The findings of this study suggested that VAH is a pre-disposing factor for atherosclerotic stenosis in the posterior circulation, and the most affected vascular segments were V1 and V4 of the vertebral artery and the middle segment of the basilar artery. Analysis of the risk factors for atherosclerotic stenosis in posterior circulation in patients with acute ischemic stroke indicated VAH as an independent risk factor.

The prevalence of VAH reported in the literature ranged from 10.8% to 43.5%.^[12–13]^ This difference could be attributed to ethnicity and the study of methodology factors. Previous studies reported that PCI was more common in patients with VAH.^[2–4,13]^ However, stroke is a heterogeneous group of diseases caused by many complex mechanisms, and the mechanism by which VAH causes ischemic stroke remains unknown. In this study, VAH pre-disposed to the development of atherosclerosis in the posterior circulation, leading to PCI.

Atherosclerosis is known to be a chronic, fibroproliferative, inflammatory, systemic disorder, mainly affecting medium- and large-sized arteries.^[14]^ All the risk factors identified for atherosclerosis were systemic disorders, and while they appear to affect all arteries, atherosclerotic plaques are focal in shape and do not show continuity.^[15,16]^ Mechanical forces affecting the arterial wall could be attributed to local factors, which in turn affect the improvement of atherosclerosis and adjusting vessel calibration and morphology.^[17]^ In addition to the systemic risk factors, fluid dynamics and artery geometry played an important role in the advancement of atherosclerosis,^[18]^ indicating that the association of local factors in vascular structures was equally important. There were 2 main forces applied to blood vessel structures by blood: transmural pressure, which is the radial force applied to the vessel wall inner row endothelial cells, and shear stress, which is tangentially applied friction according to the direction of flow. The value of endothelial shear stress (ESS) was determined by blood viscosity and friction rate. The blood flow in partially straight and uniform diameter vessels was laminar and the ESS value was normal. Physiological ESS values were demonstrated to be atheroprotective, and low or high values were atherogenic studies. Atherosclerotic plaques were also located in areas where shear stress was less or more than normal, such as branches of the arteries, lateral wall of the bifurcations, and vascular curvatures.^[19–21]^

The basilar artery originates from the confluence of the 2 vertebral arteries. Contrary to several systemic arteries, which have a tree-like branching pattern, the basilar artery is the only large artery in which 2 arterial flows unite. Initially, constant flow in a planar junction model indicated that ESS was lower forward the lateral wall of the basilar artery on the same side as the dominant vertebral artery, and this low ESS territory stretches the dimension of the basilar artery. An autopsy study conducted found that in the presence of a diameter difference between the vertebral arteries, the plaque thickness in the lateral wall of the basilar artery was greater than in the dominant vertebral artery. This difference in the basilar artery plaque thickness implicates the role of hemodynamics in the advancement of atherosclerosis.^[22]^ A study with high-resolution MRA showed that the flow characteristics (velocity and ESS distributions) in the vertebrobasilar system of healthy young adults described both the geometry and corresponding flow velocity of the system.^[23]^ Other studies revealed that blood flow dynamics described the occurrence and advancement of arteriosclerosis.^[24,25]^

Some studies suggested that the circle of Willis and its abutting arteries preserve the cerebral artery and blood-brain barrier from hemodynamic stress.^[26]^ According to this hypothesis, some variations in the circle of Willis were considered to affect the volume flow rates of the bilateral internal carotid artery and basilar artery in healthy individuals and the advancement of atherosclerosis.^[27,28]^ In this study, atherosclerotic stenosis was more common in anterior circulation in patients with VAH, which may be contributed to the variations in the circle of Willis. Angiographic evidence revealed that ethnic differences exist in the distribution of craniocervical atherosclerotic stenosis in patients with cerebral ischemia.^[29]^

Although the precise mechanism concerning how VAH pre-disposes atherosclerosis in the posterior circulation remains unknown, this study suggested that VAH pre-disposes atherosclerosis only in certain segments of the posterior circulation.

The risk factors for craniocervical atherosclerotic stenosis should be defined separately for all artery segments. To our knowledge, this was the first study that examined the relationship between atherosclerotic segments of the posterior circulation and VAH. In addition, we believed that the histological structure of the intra-cranial and cervical arteries and risk factors for atherosclerosis are different. For example, some parameters protect the intra-cranial arteries from atherosclerosis, and these protective factors could be some variations in the circle of Willis, high anti-oxidant capacity in the intra-cranial arteries, low inflammatory response, the protective effect of cerebrospinal fluid, and cerebral reactivity.^[30]^ Possible hemodynamic causes of increased posterior circulation atherosclerotic stenosis in patients with VAH include increased pressure difference in bifurcation areas in VAH patients, lower blood flow rate in hypoplastic vertebral artery, accumulation of cellular elements in a significant increase of blood viscosity, and contact of the luminal surface with LDL in the hypoplastic vessel. Elongation, increased ESS in the hypoplastic vessel, decreased ESS in the dominant vessel and basilar artery, and all these hemodynamic factors damage the endothelium.

Hemodynamic parameters associated with atherosclerosis could not be measured in vivo. Thus, to better understand the mechanism, conducting studies that examine blood flow parameters with high-resolution magnetic resonance angiography in cerebral ischemia patients with VAH.

### 4.1 Limitations of the study

This is a retrospective study that existed of only CTA. The pre-requisite for ionizing radiation and contrast medium is the main limitation. Therefore, more precise results can be achieved with a prospective study.

## Author contributions

**Data curation:** Yasemin Dinç.

**Formal analysis:** Büşra Emir.

**Funding acquisition:** Büşra Emir.

**Investigation:** Yasemin Dinç, Rifat Ozpar.

**Methodology:** Yasemin Dinç, Rifat Ozpar.

**Software:** Büşra Emir.

**Supervision:** Bahattin Hakyemez, Mustafa Bakar.

**Validation:** Rifat Ozpar.

**Writing – original draft:** Yasemin Dinç, Rifat Ozpar.

**Writing – review & editing:** Yasemin Dinç, Rifat Ozpar.
